# Effects of feeding a *Bacillus*-based probiotic on nutrient utilization and microbiota profile in the rumen and reproductive tract of beef heifers receiving a forage-based diet

**DOI:** 10.1093/jas/skaf411

**Published:** 2025-11-29

**Authors:** Izadora S De Souza, Reinaldo F Cooke, Shea J Mackey, Thainá Minela, Zachary K Seekford, Giuseppe Copani, Raphaele Gresse, Kelsey M Harvey, Ky G Pohler, João M B Vendramini, Bruno I Cappellozza

**Affiliations:** Department of Animal Science, Texas A&M University, College Station, TX 77843; Department of Animal Science, Texas A&M University, College Station, TX 77843; Department of Animal Science, Texas A&M University, College Station, TX 77843; Department of Animal Science, Texas A&M University, College Station, TX 77843; Department of Animal Science, Texas A&M University, College Station, TX 77843; Novonesis, Lyngby 2800, Denmark; Novonesis, Lyngby 2800, Denmark; Prairie Research Unit, Mississippi State University, Prairie, MS 39756; Department of Animal Science, Texas A&M University, College Station, TX 77843; Stephenville Research and Extension Center, Texas A&M AgriLife Research, Stephenville, TX 76401; Novonesis, Lyngby 2800, Denmark

**Keywords:** *Bacillus* spp, beef heifer, microbiota, nutrient degradation, rumen fermentation

## Abstract

This experiment evaluated the effects of feeding a *Bacillus*-based probiotic on rumen fermentation traits, apparent nutrient digestibility, microbiota profile in the rumen and reproductive tract of beef heifers receiving a forage-based diet. Sixteen rumen-cannulated, pubertal Angus-influenced heifers were used in a crossover design (35-d periods). Treatments included hay and concentrate (**CON;** *n* = 16) or CON with the addition of 3 g/heifer daily of *Bacillus*-based probiotic (**BAC**; *n* = 16). Blood samples were collected on days 0, 7, 14, 21, 28, and 35. Rumen fluid samples were collected on days 0, 14, and 28. Vaginal and uterine swabs were collected on day 28. On day 32 of each period, ruminal liquid fill, liquid dilution rate, forage dry matter (**DM**) and neutral detergent fiber (**NDF**) disappearance were assessed for 96 h. Apparent total-tract digestibility (**ATTD**) of nutrients was calculated using fecal samples collected from days 32 to 35. Data were analyzed using heifer as the experimental unit, and results from day 0 as covariate when appropriate. Ruminal fluid pH and ammonia concentrations did not differ between treatments (*P *≥ 0.28). Acetate proportion in the ruminal fluid was reduced (*P *< 0.01), whereas rumen propionate, butyrate, and iso-valerate proportions were increased (*P *≤ 0.05) in BAC heifers. No treatment differences were observed (*P *≥ 0.14) for rumen fluid phyla. Relative abundances of the genera *Bacteroides*, *Pedobacter*, and *Clostridium* were less (*P *≤ 0.04) in the rumen fluid of BAC heifers. Rumen liquid volume and dilution rate were not affected (*P *≥ 0.63) by treatments. Heifers assigned to BAC had greater (*P *≤ 0.04) effective ruminal degradability of DM and NDF, as well as ATTD of DM, CP, and NDF. Mean plasma glucose and insulin concentrations were greater (*P *≤ 0.04) while mean blood urea-N concentration was less (*P *< 0.01) in BAC heifers. Plasma IGF-I concentration was greater (*P *= 0.04) in BAC heifers on day 21 (treatment × day; *P *= 0.04). Heifers receiving BAC had reduced (*P *= 0.05) abundance of the phylum Bacteroidetes, and reduced (*P *≤ 0.05) abundance of the genera *Pedobacter* and *Bacteroides* in vaginal swabs. No treatment effects were observed (*P *≥ 0.29) for phyla or genera relative abundances in uterine swabs. Supplementing a *Bacillus*-based probiotic improved nutrient utilization, rumen fermentation traits, and physiological responses of beef heifers consuming a forage-based diet, with effects on the reproductive tract limited to the vaginal microbiota.

## Teaser

Supplementing probiotics to beef cattle can improve digestibility of forage nutrients, and modulate the microbiota of the female reproductive tract. Hence, this experiment evaluated how a *Bacillus*-based probiotic impact ruminal and overall nutrient utilization, physiological responses, and reproductive tract microbiota of beef heifers fed a forage-based diet.

## Introduction

Forages are the main source of nutrients for most cow-calf production systems, including growing cattle and the mature cowherd. Nutritional strategies to complement forage deficiencies or improve the use of forage nutrients are often required, given the seasonal variations in forage quality and quantity (Kunkle et al., 2000). One example is the use of *Bacillus*-based probiotics, which optimize nutrient digestibility through their enzymatic ability (Schallmey et al., 2004; Luise et al., 2022). Previous research described an increased nutrient degradation of forage-based feedstuffs in vitro (Pan et al., 2022; Cappellozza et al., 2023) and in vivo (Eyre et al., 2025; Limede et al., 2025) with the use of *Bacillus*-based probiotics, although most of these studies evaluated C3 forages. Supplementing a *Bacillus*-based probiotic improved body condition score in beef heifers grazing C4 forages (Izquierdo et al., 2024), and authors associated these results with enhanced enzyme production in the gastrointestinal tract. Research is still warranted to evaluate the role of *Bacillus*-based probiotics in improving nutrient digestibility of diets based in C4 forages, which predominate in tropical/subtropical regions of the US and the world (Cooke et al., 2020).

*Bacillus*-based probiotics modulate rumen microbiota (Limede et al., 2025), which can impact the microbiota profile of the reproductive tract and fertility of beef females (Ault et al., 2019a; Ault-Seay et al., 2022; Pickett et al., 2022). *Bacillus licheniformis* and *B. subtilis* were found in uterine cultures of dairy cows without endometritis, while their presence was not detected in cows with metritis (Ballas et al., 2021). No research has evaluated the impacts of feeding a *Bacillus*-based probiotic on microbiota profile in the reproductive tract of beef females. Based on this rationale, we hypothesized that a *Bacillus*-based probiotic will improve nutrient utilization and impact the reproductive tract microbiota of beef heifers offered a diet based on C4 forages. To test this hypothesis, this experiment evaluated ruminal fermentation traits, ruminal nutrient degradation, apparent total-tract digestibility, and physiological responses, as well as ruminal, vaginal, and uterine microbiota profile in beef heifers offered a bermudagrass-based diet.

## Materials and Methods

This experiment was conducted at the Texas A&M—Nutrition & Physiology Center (College Station, TX). All animals were cared for in accordance with acceptable practices and experimental protocols reviewed and approved by the Texas A&M AgriLife Research, Agriculture Animal Care and Use Committee (#2024-0232).

### Animals and treatments

Sixteen ruminally cannulated, pubertal, nulliparous, nonpregnant Angus (75%) × Brahman (25%) heifers were used. All heifers were around 15 mo of age and puberty status was determined by a pretrial reproductive tract score that was conducted by a trained technician (Holm et al., 2009). All heifers had a score ≥ 4 and were assigned to an estrous synchronization protocol (Larson et al., 2006) so that ovulation occurred 3 d prior to the beginning of the experiment. Heifer unshrunk body weight (**BW**) was recorded for three consecutive days prior to the beginning of the experiment, and averaged to represent pre-trial BW (299 ± 3 kg). Heifers were ranked by pre-trial BW and assigned to two groups of eight heifers each, and groups had equivalent pre-trial BW. Groups were enrolled in a crossover design containing two periods of 35 d, and a 14-d washout interval between periods. Heifers were housed in an enclosed barn in individual pens (2 × 4 m) during each period (day 0 to 35). Heifers were maintained as a single group in a drylot pen (20 × 20 m) for the initial 10 d of the washout interval, and returned to individual pens for the remaining 4 d. During each period, heifers had ad libitum access to water and bermudagrass hay (*Cynodon dactylon*) and were fed of a corn-based concentrate (Table 1) at 0700 h. Hay and concentrate were offered in separate feed bunks. Formulation and feeding rate of the concentrate were selected to yield an average daily gain (**ADG**) of approximately 550 g/d (NASEM, 2016), according to nutritive value of the forage used herein (Table 1). Melengestrol acetate (**MGA**) was added to the concentrate from days 14 to 35 of each period to suppress ovulation (Pickett et al., 2022) due to shifts in the abundance of bacterial communities throughout the estrous cycle (Ault et al., 2019a, 2019b; Quereda et al., 2020). Heifers had ad libitum access to water and bermudagrass hay during the washout period. During the washout period, heifers were again assigned to an estrous synchronization protocol (Larson et al., 2006) so that ovulation occurred 3 d prior to the beginning of second period.

**Table 1.** Nutrient profile (dry matter basis) of feed ingredients offered to heifers^1^^,^^2^

Item	Hay	Corn	Cottonseed meal	MGA supplement	**Concentrate (day** −**3 to 13)**	Concentrate (day 14 to 35)
**Net energy for maintenance,^3^ Mcal/kg**	1.10	2.29	1.63	1.36	2.03	2.06
**Net energy for gain,^3^ Mcal/kg**	0.53	1.58	1.03	0.79	1.38	1.35
**Crude protein, %**	7.30	8.02	52.2	2.80	17.3	14.5
**Starch, %**	5.20	67.3	0.60	5.75	49.4	41.0
**Acid detergent fiber, %**	37.3	2.96	12.3	38.8	4.86	11.4
**Neutral detergent fiber, %**	71.9	16.4	25.2	47.2	17.5	23.3
**Indigestible neutral detergent fiber, %**	27.5	0.840	4.27	53.4	1.54	11.7

1 Analyzed via wet chemistry procedures by a commercial laboratory (Dairy One Forage Laboratory, Ithaca, NY).

2 Heifers had ad libitum access to bermudagrass hay (*Cynodon dactylon*) and were fed 0.940 kg/d [dry matter (**DM**) basis] of a concentrate (73.3% corn, 21.9% cottonseed meal, and 4.8% of vitamin + mineral mix; DM basis) from d -3 to 13, and 1.16 kg/d (DM basis) of a concentrate (59.2% corn, 17.6% cottonseed meal, 19.3% of a supplement delivering melengestrol acetate [**MGA**], and 3.9% of vitamin + mineral mix; DM basis) from days 14 to 35 of the experiment. The vitamin + mineral mix contained 21% Ca, 0.01% P, 21% NaCl, 0.20% K, 0.10% Mg, 0.045% Cu, 0.001% Se, 0.280% Zn, 220,000 IU/kg of vitamin A, 19,800 IU/kg of vitamin D3, and 3,500 IU/kg of vitamin E (Anipro Xtraperformance Feeds, College Station, TX).

3 Calculated with the following equations (NASEM, 2016): Net energy for maintenance = (1.37 × ME)—(0.138 × ME^2^) + (0.0105 × ME^3^)—(1.12); Net energy for gain = (1.42 × ME) – (0.174 × ME^2^) + (0.0122 × ME^3^) – (0.165); ME = (0.82 × DE); 1 kg of TDN = 4.4 Mcal of DE. Given that ME = metabolizable energy, DE = digestible energy, TDN = total digestible nutrients calculated based on Weiss et al. (1992).

At the beginning of the first period, groups were assigned to receive (as fed basis) one of two treatments: 1) no probiotic supplementation (**CON**) or 2) 3 g/heifer per day of a *Bacillus*-based probiotic (Bovacillus, Novonesis, Milwaukee, WI, USA; **BAC**) from days 5 to 35. Groups were assigned to receive treatments during period 2 in a crossover fashion, resulting in *n* = 16 heifers/treatment. The inclusion rate of the *Bacillus*-based probiotic was based on manufacturer’s recommendations for growing cattle and Izquierdo et al. (2024), including a proprietary composition to provide 2.20 × 10^9^ CFU/g of *B. subtilis* 810 and *B. licheniformis* 809 bacteria. The proper dose of the *Bacillus*-based probiotic was mixed daily with the concentrate during each period (from days 5 to 35), which was completely consumed within 30 min after feeding.

### Sampling and laboratorial analysis

#### Feed samples

Samples of feed ingredients were collected prior to the beginning of each period, pooled across all periods, and analyzed for nutrient content by a commercial laboratory (Dairy One Forage Laboratory, Ithaca, NY). Sample DM and organic matter (**OM**) were calculated as in NASEM (2016). Samples were also analyzed by wet chemistry procedures for concentrations of crude protein (**CP**; method 984.13; AOAC, 2006), acid detergent fiber (**ADF**; method 973.18 modified for use in an Ankom 200 fiber analyzer, Ankom Technology Corp., Fairport, NY; AOAC, 2006), neutral detergent fiber (**NDF**) using α-amylase and sodium sulfite (Van Soest et al., 1991; modified for use in an Ankom 200 fiber analyzer, Ankom Technology Corp.), starch (YSI 2700 SELECT Biochemistry Analyzer; YSI Inc., Yellow Springs, OH), and indigestible neutral detergent fiber (**iNDF**) as described by Cole et al. (2011) with modifications proposed by Krizsan and Huhtanen (2013). More specifically, iNDF was analyzed using ground samples (2 mm) that were weighed (0.5 g) into F57 filter bags (Ankom Technology, Macedon, NY), and incubated at 39°C using a 4:1 ratio of McDougall’s buffer: rumen fluid in a Daisy^II^ incubator (Ankom Technology Corp.) for 288 h. After incubation, samples were rinsed and analyzed for NDF concentration as previously described (Van Soest et al., 1991). Calculations for net energy for maintenance and gain used equations from the NASEM (2016). Nutritional composition and profile of dietary treatments are described in Table 1.

#### Heifer BW and intake

Heifer unshrunk BW was recorded on days 0, 1, and 2 of each period and averaged for initial BW. Final BW was calculated by averaging heifer unshrunk BW on days 33, 34, and 35 of each period. Hay intake was evaluated daily from days 0 to 35 by weighing and collecting samples of the offered and non-consumed hay, and drying samples for 96 h at 50 °C in forced-air ovens for DM calculation. Feed intake was calculated according to daily hay and concentrate intake. Heifer ADG was calculated using initial and final BW of each period. Gain-to-feed ratio (**G:F**) was calculated according to total feed intake and total BW gain of each heifer within each period.

#### Physiological responses

Blood samples were collected from all heifers on days 0, 7, 14, 21, 28, and 35 of each period at 4 h post-treatment feeding. Blood was collected via jugular venipuncture into commercial blood collection tubes (Vacutainer, 10 mL; Becton Dickinson, Franklin Lakes, NJ) containing freeze-dried sodium heparin for plasma extraction. Blood samples were placed immediately on ice after collection, centrifuged (2,500 × *g* for 30 min; 4 °C) for plasma harvest, and stored at −80°C within 24 h. All plasma samples were analyzed for concentrations of glucose and urea-N (respons 910VET Veterinary Chemistry Analyzer; DiaSys Diagnostic Systems USA LLC, Wixom, MI; Colombo et al., 2021), insulin (#PI-12K; EMD Millipore Corporation, Billerica, MA; Kaufman et al., 2018), and insulin-like growth factor (**IGF**)-I (#SG100; R&D Systems, Inc., Minneapolis, MN; Cooke et al., 2012). The intra- and inter-assay CVs for all analyses were ≤ 9.1%.

#### Rumen fluid, uterine, and vaginal swab collection

Rumen fluid samples were collected via rumen cannula from all heifers concurrently with blood samplings on days 0, 14, and 28 of each period at 4 h post-treatment feeding (Pickett et al., 2022). The pH of the rumen samples was immediately measured using a pH portable meter (Orion Star; Thermo Fisher Scientific Inc., Waltham, MA). Rumen samples were strained through eight layers of cheesecloth for fluid extraction, which was stored into individual stainless-steel thermoses to maintain both temperature and an anaerobic environment, transported to the laboratory, and stored at −80°C until further processing. A 5-mL subsample of each rumen fluid sample was transferred into individual falcon tubes containing 1 mL of 25% metaphosphoric acid and stored at –20 °C on the same day of collection for ammonia and VFA analysis (Cappellozza et al., 2013). Another 5-mL subsample of the rumen fluid was immediately snap-frozen in liquid N for microbiota analysis as in Pickett et al. (2022).

Uterine and vaginal swabs were collected on day 28 of each period at 4 h post-feeding as previously described (Moore et al., 2023). Briefly, the perineal area was sanitized with antiseptic solution and dried with paper towels. A bovine cytology brush (Minitüb GmbH, Germany) was placed in the vagina. The cytobrush was extended beyond the outer sheath and rotated once to sample the contents of the vagina, retracted into the outer sheath and removed from the vagina. The uterine sample was collected immediately after the vaginal sampling. A steel catheter enclosed within a plastic chemise was placed in the vagina. The chemise was pierced and the catheter was passed through the cervix to the uterine body. A bovine uterine cytology brush was guided through the catheter, extended beyond the outer sheath and rotated once to sample the contents of the uterus. The cytobrush was retracted into the outer sheath and removed from the uterus together with the steel catheter. Each cytobrush was cut after sampling the vagina or uterus, placed in a 15-mL sterile conical tube (Sarstedt Ltd, Ireland), immediately snapped-frozen in liquid N for microbiota analysis as in Pickett et al. (2022).

#### Ruminal kinetic dynamics and in situ forage disappearance

On day 32 of each period and immediately after treatment administration, Dacron bags (50 ± 10 μm pore size and 10 by 20 cm bag size; Ankom Technology Corp.) containing 4 g (DM basis) of ground dietary bermudagrass hay (2-mm screen; Wiley Mill, Model 4; Arthur H. Thomas, Philadelphia, PA) were suspended into the ruminal ventral sac of each heifer and incubated in triplicates for 0, 2, 4, 6, 8, 12, 24, 36, 48, 72, and 96 h. Before ruminal incubation, all bags were soaked in warm water (39°C) for 15 min. After ruminal incubation, bags were washed repeatedly with running water until the rinse water was colorless and dried for 96 h at 50°C in a forced-air oven. The 0-h bags were not incubated in the rumen but were subjected to the same soaking, rinsing, and drying procedures applied to the ruminally incubated bags. Dried samples were weighed for residual DM determination, and triplicates were combined and analyzed for NDF (Van Soest et al., 1991) with sodium sulfite and heat stable amylase (Ankom 200 Fiber Analyzer; Ankom Technology Corp.). Effective degradability of hay DM and NDF were calculated fixing ruminal passage rate at 0.035/h (Scarbrough et al., 2006) and the model from Ørskov and McDonald (1979).

Immediately after Dacron bags were suspended into the rumen, all heifers were intra-ruminally pulse-dosed with 5 g of Co-EDTA in a 150-mL aqueous solution (Udén et al., 1980). The Co marker was administered throughout the rumen by injecting through a stainless-steel probe with a perforated tip. Ruminal fluid (approximately 100 mL) was collected by suction strainer (Raun and Burroughs, 1962) immediately prior to dosing (0 h) and at 2, 4, 6, 8, 12, 24, 36, 48, 72, and 96 h after dosing. Twenty milliliters of the ruminal fluid were stored (−20°C) for analysis of Co concentration by atomic absorption using an air-acetylene flame (Model 351 atomic absorption spectrophotometry, Instrumentation Laboratory, Inc., Lexington, MA). Ruminal liquid fill and liquid dilution rate were estimated by regression of the natural logarithm of Co concentration against sampling time as described by Warner and Stacy (1968).

#### Apparent total-tract nutrient digestibility (ATTD)

Fecal grab samples were collected from days 32 to 35 of each heifer at 12-h intervals. Samples were dried for 168 h at 50°C in a forced-air oven, composited within each period, and used to calculate ATTD of DM, OM, CP, NDF, and starch using iNDF as internal marker and the equations described by Ferraretto et al. (2015): *aTTD (% of nutrient intake) = 100 − [(dietary marker concentration/fecal marker concentration) × (fecal nutrient concentration/dietary nutrient concentration)].* Concentrations of OM, CP, NDF, starch, and iNDF in feces were determined as described for feed ingredients.

### Statistical analysis

Heifer was considered the experimental unit for all analyses. All data were analyzed with the MIXED procedure of SAS (SAS Inst. Inc., Cary, NC), using heifer as random variable. The model statement used for feed intake, blood and rumen fluid variables contained the effects of treatment, day, the treatment × day interaction, and period as independent variable. The specified term for these repeated statements was day, with heifer (treatment × period) as the subject, and autoregressive as covariance structure based on the Akaike information criterion. Results from blood and ruminal fluid variables collected on day 0, and average feed intake from days 0 to 4, were used as independent covariate in each respectively analysis. The model statement used for all other variables contained the effects of treatment and period as independent variable. All results are reported as least square means, or covariately adjusted least square means when model contained results prior to treatment administration. Significance was set at *P *≤ 0.05 and tendencies were determined if *P *> 0.05 and ≤ 0.10. Repeated measures are reported according to main treatment effect if the treatment × day interaction was *P *> 0.10.

## Results

Heifer initial BW did not differ (*P *= 0.94) between treatments as expected (Table 2), and no treatment effects were detected (*P *≥ 0.59) for final BW, ADG, feed intake, or G:F (Table 2). No treatment × day interactions were detected (*P *≥ 0.38) for plasma glucose, urea-N, or insulin concentrations (Table 3). Heifers assigned to BAC had greater (*P *≤ 0.04) mean plasma glucose and insulin, and reduced (*P *< 0.01) mean plasma urea-N concentrations compared to CON heifers (Table 3). A treatment × day interaction was observed (*P *= 0.04) for plasma IGF-I concentration, which was greater (*P *= 0.04) for BAC heifers on day 21 compared with CON heifers (Table 3).

**Table 2.** Growth performance of rumen-cannulated forage-fed heifers supplemented or not (**CON**; *n* = 16 heifers) with a *Bacillus*-based probiotic (**BAC**; *n* = 16 heifers)^1^^,^^2^

Item	BAC	CON	SEM	*P*-value
**Initial BW, kg**	303.5	302.5	9.7	0.94
**Final BW, kg**	323.7	322.3	10.7	0.93
** ADG, kg/day**	0.561	0.550	0.055	0.89
**Feed intake, kg/day**				
** Hay only**	6.29	6.20	0.11	0.59
** Hay + concentrate**	7.40	7.31	0.11	0.59
**Gain to feed, kg/kg**	0.076	0.077	0.007	0.91

1 Heifers were assigned to receive a concentrate containing either: 1) *Bacillus licheniformis* 809 and *B. subtilis* 810 probiotic (**BAC**; 3 g/heifer daily of Bovacillus; Novonesis, Milwaukee, WI, USA) or 2) no additive (**CON**). Treatments were offered from days 5 to 35 of the experiment, but all heifers received the CON concentrate from day −3 to 4.

2 Initial body weight (**BW**) was calculated by averaging heifer BW on day 0, 1, and 2. Final BW was calculated by averaging heifer BW on day 34, 35, and 36. Hay intake was evaluated daily (day −3 to 35), and heifers entirely consumed the concentrate within 30 min after feeding (0700 h). Feed intake from day −3 to 4 of each heifer was averaged and used as covariate in each respective analysis. Heifer average daily gain (**ADG**) was calculated using initial and final BW, whereas gain to feed ratio was calculated according to total feed intake and BW gain of each heifer. No treatment × day interaction was detected for hay and hay + concentrate intake (*P *≥ 0.41); hence, feed intake results are reported according to main treatment effect.

**Table 3.** Plasma hormones and metabolites in rumen-cannulated forage-fed heifers supplemented or not (**CON**; *n* = 16 heifers) with a *Bacillus*-based probiotic (**BAC**; *n* = 16 heifers)^1^^,^^2^

Item	BAC	CON	SEM	*P*-value
**Glucose, mg/dL**	72.7	68.9	1.0	0.02
**Blood urea N, mg/dL**	5.63	6.62	0.2	<0.01
**Insulin, pg/mL**	16.4	10.8	1.8	0.04
**Insulin-like growth factor I, ng/mL**				
** Day 7**	115	109	8	0.54
** Day 14**	96.5	113.2	8	0.15
** Day 21**	139	115	8	0.04
** Day 28**	136	140	8	0.74
** Day 35**	127	141	8	0.24

1 Heifers were assigned to receive a concentrate containing either: 1) *Bacillus licheniformis* 809 and *B. subtilis* 810 probiotic (**BAC**; 3 g/heifer daily of Bovacillus; Milwaukee, WI, USA) or 2) no additive (**CON**). Supplement treatments were offered from days 5 to 35 of the experiment.

2 Blood samples were collected on days 0, 14, and 28 of the experiment, 4 h after the concentrate feeding of the day. Results from day 0 were used as covariate for each respective analysis. A treatment × day interaction was detected (*P *≤ 0.04) for insulin-like growth factor I, but not for the other parameters (*P *≥ 0.38) and their results are reported according to main treatment effects.

No treatment × day interactions were detected (*P *≥ 0.32) for rumen fermentation responses (Table 4). Ruminal fluid pH and ammonia concentrations did not differ (*P *≥ 0.28) between treatments. Heifers receiving BAC had reduced (*P *= 0.02) valerate concentration and acetate: propionate ratio in the rumen fluid compared with CON heifers. No treatment differences were detected (*P *≥ 0.25) for concentrations the other VFAs or total VFA concentration in the rumen fluid (Table 4). When analyzed as % of total VFA, the acetate proportion was less (*P *< 0.01) whereas proportions of propionate, butyrate, and iso-valerate were greater (*P *≤ 0.05) in the rumen fluid of BAC heifers compared with CON heifers (Table 4).

**Table 4.** Rumen fermentation responses in rumen-cannulated forage-fed heifers supplemented or not (**CON**; *n* = 16 heifers) with a *Bacillus*-based probiotic (**BAC**; *n* = 16 heifers)^1^^,^^2^

Item	BAC	CON	SEM	*P*-value
**Rumen fluid pH**	6.30	6.30	0.04	0.98
**Rumen ammonia, m*M***	0.655	0.840	0.112	0.28
**Rumen fluid VFA, m*M***				
** Acetate**	56.2	63.6	4.3	0.27
** Propionate**	14.8	14.7	0.8	0.97
** Butyrate**	10.1	10.3	0.4	0.70
** Iso-valerate**	0.723	0.679	0.035	0.38
** Iso-butyrate**	0.625	0.654	0.016	0.25
** Valerate**	0.958	1.05	0.028	0.02
** Acetate: propionate ratio**	3.80	4.25	0.12	0.02
** Total**	83.7	90.7	5.6	0.41
**Rumen fluid VFA, % of total VFA**				
** Acetate**	66.8	69.5	0.6	<0.01
** Propionate**	17.7	16.5	0.4	0.05
** Butyrate**	12.5	11.3	0.4	0.04
** Iso-valerate**	0.922	0.742	0.048	0.02
** Iso-butyrate**	0.794	0.722	0.036	0.23
** Valerate**	1.22	1.14	0.04	0.25

1 Heifers were assigned to receive a concentrate containing either: 1) *Bacillus licheniformis* 809 and *B. subtilis* 810 probiotic (**BAC**; 3 g/heifer daily of Bovacillus; Novonesis, Milwaukee, WI, USA) or 2) no additive (**CON**). Supplement treatments were offered from day 5 to 35 of the experiment.

2 Ruminal fluid samples were collected on day 0, 14, and 28 of each period, 4 h after the concentrate feeding of the day. The pH of the rumen samples was immediately measured using portable a meter (Traceable™; Thermo Fisher Scientific, Waltham, MA). A 5-mL subsample of each rumen fluid sample was transferred into individual falcon tubes containing 1 mL of 25% metaphosphoric acid for analyses of volatile fatty acids (**VFA**) and ammonia content (Cappellozza et al., 2013). Results from day 0 were used as covariate for each respective analysis. No treatment × day interactions were detected (*P *≥ 0.32) for the parameters reported herein; therefore, results are reported according to main treatment effects.

Rumen liquid volume and dilution rate were not affected (*P *≥ 0.63) by treatments (Table 5). Heifers assigned to BAC tended (*P *≤ 0.08) to have greater ruminal disappearance rates of DM and NDF, and had greater (*P *≤ 0.05) ruminal effective degradability of DM and NDF compared with CON heifers (Table 5). From days 32 to 35 of each period, daily intake of DM, OM, CP, NDF, starch, and iNDF did not differ (*P *= 0.72) between treatments (Table 6). Heifers assigned to BAC had greater (*P *≤ 0.04) ATTD of DM, CP, and NDF (Table 6), and tended (*P *≤ 0.09) to have greater ATTD of OM and ADF compared with CON heifers (Table 6). No treatment differences were detected for ATTD of starch (*P *= 0.37).

**Table 5.** Rumen liquid dilution rate, rumen liquid volume, and ruminal in situ disappearance parameters of bermudagrass hay (*Cynodon dactylon*) in rumen-cannulated forage-fed heifers supplemented or not (**CON**; *n* = 16 heifers) with a *Bacillus*-based probiotic (**BAC**; *n* = 16 heifers)^1^

Item	BAC	CON	SEM	*P*-value
**Liquid dilution rate,^2^ %/h**	8.57	8.84	0.38	0.63
**Liquid volume,^2^ mL/kg of BW**	186	183	14	0.87
**Hay disappearance rate,^3^ %/h**				
** Dry matter**	3.36	2.65	0.27	0.08
** Neutral detergent fiber**	3.90	3.10	0.30	0.07
**Hay effective degradability,^4^ %**				
** Dry matter**	42.3	40.2	0.7	0.05
** Neutral detergent fiber**	52.0	50.1	0.5	0.03

1 Heifers were assigned to receive a concentrate containing either: 1) *Bacillus licheniformis* 809 and *B. subtilis* 810 probiotic (**BAC**; 3 g/heifer daily of Bovacillus; Novonesis, Milwaukee, WI, USA) or 2) no additive (**CON**). Supplement treatments were offered from day 5 to 35 of the experiment.

2 Liquid dilution rate and volume were evaluated via intra-ruminal Co-EDTA pulse-dose on day 32 prior to concentrate feeding, and rumen fluid collected at 0, 2, 4, 6, 8, 12, 24, 36, 48, 72, and 96 h relative to Co-EDTA infusion (Cappellozza et al., 2013).

3 Hay disappearance rate was evaluated by suspending Dacron bags containing 4 g of hay on day 32 prior to concentrate feeding, and incubating by 0, 2, 4, 6, 8, 12, 24, 36, 48, 72, and 96 h.

4 Effective degradability was calculated by fixing ruminal passage rate at 0.035/h (Scarbrough et al., 2006) and the model proposed by Ørskov and McDonald (1979).

**Table 6.** Nutrient intake and apparent total-tract digestibility in rumen-cannulated forage-fed heifers supplemented or not (**CON**; *n* = 16 heifers) with a *Bacillus*-based probiotic (**BAC**; *n* = 16 heifers)^1^^,^^2^

Item	BAC	CON	SEM	*P*-value
**Nutrient intake, kg/d**				
** Dry matter**	7.15	6.97	0.34	0.72
** Organic matter**	6.18	6.03	0.30	0.72
** Crude protein**	0.605	0.592	0.02	0.72
** Acid detergent fiber**	2.36	2.29	0.12	0.72
** Neutral detergent fiber**	4.57	4.44	0.24	0.72
** Starch**	0.789	0.780	0.02	0.72
** Indigestible neutral detergent fiber**	1.78	1.73	0.09	0.72
**Apparent total-tract digestibility, % of intake**				
** Dry matter**	55.0	53.0	0.5	0.02
** Organic matter**	57.2	55.7	0.6	0.08
** Crude protein**	54.9	51.1	0.7	<0.01
** Neutral detergent fiber**	53.4	51.3	0.7	0.04
** Acid detergent fiber**	38.1	35.9	0.8	0.09
** Starch**	90.1	88.7	1.1	0.37

1 Heifers were assigned to receive a concentrate containing either: 1) *Bacillus licheniformis* 809 and *B. subtilis* 810 probiotic (**BAC**; 3 g/heifer daily of Bovacillus; Novonesis, Milwaukee, WI, USA) or 2) no additive (**CON**). Supplement treatments were offered from day 5 to 35 of the experiment.

2 Apparent total-tract digestibility of nutrients was calculated by collecting fecal grab samples collected at 12-h intervals beginning on day 32 prior to concentrate (h 0) feeding until day 35 (h 96), according to Ferraretto et al. (2015) and using indigestible neutral detergent fiber. Nutrient intake was calculated based on hay and concentrate intake from day 32 to 35, and the nutritional analysis of all feed ingredients.

No treatment × day interactions were detected (*P *≥ 0.23) for microbiota assessments in the ruminal fluid (Table 7). Relative abundance of bacterial phyla and phyla Shannon diversity (**SD**) in the ruminal fluid were not affected (*P *≥ 0.14) by treatments (Table 7). Heifers assigned to BAC had less (*P *= 0.04) relative abundance of the genera *Bacteroides*, *Pedobacter*, and *Ruminococcus* compared in the ruminal fluid with CON heifers (Table 7). Relative abundance of all other bacterial genera and genera SD in the ruminal fluid did not differ (*P *≥ 0.12) between treatments (Table 7).

**Table 7.** Bacterial composition (relative abundance, %) and diversity [Shannon diversity (**SD;**  Kim et al., 2017)] in the rumen fluid of rumen-cannulated forage-fed heifers supplemented or not (**CON**; *n* = 16 heifers) with a *Bacillus*-based probiotic (**BAC**; *n* = 16 heifers)^1^^,^^2^

Item	BAC	CON	SEM	*P*-value
**Bacterial phyla**				
** Bacteroidetes**	51.7	50.4	0.9	0.32
** Firmicutes**	29.5	31.0	0.7	0.14
** Proteobacteria**	11.0	10.9	0.4	0.92
** Euryarchaeota**	2.35	2.24	0.15	0.60
** Tenericutes**	1.50	1.56	0.10	0.72
** Spirochaetes**	1.47	1.47	0.10	0.98
** SD index**	0.930	0.926	0.012	0.82
**Bacterial genera**				
***Prevotella***	23.2	21.2	0.9	0.15
***Bacteroides***	9.62	10.5	0.27	0.04
***Pedobacter***	7.72	8.84	0.27	<0.01
***Paludibacter***	5.09	3.80	0.52	0.12
***Caloramator***	4.33	4.31	0.12	0.93
***Dysgonomonas***	3.17	3.24	0.22	0.82
***Clostridium***	2.79	2.91	0.12	0.50
***Ruminococcus***	2.67	3.43	0.22	0.04
***Methanobrevibacter***	2.67	2.60	0.17	0.73
***Oscillospira***	2.57	2.96	0.17	0.13
***Blautia***	2.47	2.60	0.17	0.65
***Butyrivibrio***	2.19	2.15	0.12	0.81
***Treponema***	1.70	1.72	0.12	0.90
***Succiniclasticum***	1.65	1.62	0.10	0.83
***Thalassospira***	1.63	1.51	0.12	0.51
***Weissella***	1.48	1.31	0.17	0.48
***Porphyromonas***	1.42	1.72	0.17	0.24
***Paraprevotella***	1.30	1.36	0.07	0.55
***Streptococcus***	1.24	1.22	0.18	0.93
***Selenomonas***	1.22	0.959	0.163	0.28
***Thermicanus***	1.15	1.16	0.04	0.95
***Alkaliphilus***	1.14	1.58	0.15	0.12
** SD index**	3.32	3.37	0.03	0.36

Only bacterial phyla and genera with relative abundance above 1% are reported.

1 Heifers were assigned to receive a concentrate containing either: 1) *Bacillus licheniformis* 809 and *B. subtilis* 810 probiotic (**BAC**; 3 g/heifer daily of Bovacillus; Novonesis, Milwaukee, WI, USA) or 2) no additive (**CON**). Supplement treatments were offered from day 5 to 35 of the experiment.

2 Ruminal fluid samples were collected on days 0, 14, and 28 of the experiment, 4 h after the concentrate feeding of the day. Results from day 0 were used as covariate for each respective analysis. No treatment × day interactions were detected (*P *≥ 0.23) for the parameters reported herein; therefore, results are reported according to main treatment effects.

Relative abundance of the phylum Bacteroidetes was less (*P *= 0.05) in vaginal swabs samples of BAC heifers compared with CON (Table 8). No other treatment effects were detected (*P *≥ 0.11) for bacterial phyla and phyla SD in vaginal samples (Table 8). Heifers assigned to BAC had less (*P ≤ *0.05) relative abundance of the genera *Pedobacter* and *Bacteroides*, and tended (*P *= 0.08) to have less relative abundance of the genus *Caloramator* in vaginal samples compared with CON heifers (Table 8). In turn, relative abundance of the genus *Corynebacterium* tended (*P *= 0.07) to be greater in BAC heifers compared with CON heifers in vaginal samples (Table 8). No treatment effects were detected (*P *≥ 0.29) for relative abundance of all bacterial phyla and genera, as well as phyla and genera SD, in uterine swab samples (Table 9).

**Table 8.** Bacterial composition (relative abundance, %) and diversity [Shannon diversity (**SD;**  Kim et al., 2017)] in the vagina of rumen-cannulated forage-fed heifers supplemented or not (**CON**; n = 16 heifers) with a *Bacillus*-based probiotic (**BAC**; n = 16 heifers)^1^^,^^2^

Item	BAC	CON	SEM	*P*-value
**Bacterial phyla**				
** Firmicutes**	48.8	46.6	4.7	0.74
** Bacteroidetes**	12.7	21.0	2.7	0.05
** Tenericutes**	17.3	17.7	7.7	0.97
** Proteobacteria**	8.12	5.59	1.70	0.31
** Actinobacteria**	8.50	4.89	1.50	0.11
** Euryarchaeota**	1.80	1.51	0.52	0.69
** SD index**	1.34	1.37	0.06	0.73
**Bacterial genera**				
***Ureaplasma***	18.2	16.3	7.7	0.86
***Corynebacterium***	7.29	3.39	1.43	0.07
***Ruminococcus***	4.92	7.73	1.46	0.19
***Campylobacter***	4.92	0.99	1.68	0.12
***Streptococcus***	4.37	2.52	1.10	0.26
***Facklamia***	4.01	1.65	1.17	0.17
***Porphyromonas***	3.54	1.87	1.19	0.34
***Clostridium***	3.36	4.18	0.60	0.35
***Helcococcus***	3.24	0.965	0.92	0.10
***Pedobacter***	3.20	6.55	1.13	0.05
***Bacteroides***	2.76	5.86	0.91	0.03
***Blautia***	2.73	3.48	0.61	0.39
***Sarcina***	2.32	1.43	0.72	0.40
***Turicibacter***	2.16	1.37	0.56	0.34
***Methanobrevibacter***	1.90	2.08	0.63	0.84
***Caloramator***	1.75	2.63	0.33	0.08
***Butyrivibrio***	1.66	2.03	0.40	0.52
***Slackia***	1.23	1.51	0.30	0.53
***Oscillospira***	1.18	1.80	0.29	0.15
** SD index**	3.25	3.49	0.20	0.40

Only bacterial phyla and genera with relative abundance above 1% are reported.

1 Heifers were assigned to receive a concentrate containing either: 1) *Bacillus licheniformis* 809 and *B. subtilis* 810 probiotic (**BAC**; 3 g/heifer daily of Bovacillus; Novonesis, Milwaukee, WI, USA) or 2) no additive (**CON**). Supplement treatments were offered from day 5 to 35 of the experiment.

2 Vaginal swabs were collected on day 28, 4 h after the concentrate feeding of the day as in Moore et al. (2023).

**Table 9.** Bacterial composition (relative abundance, %) and diversity [Shannon diversity (**SD;**  Kim et al., 2017)] in the uterus of rumen-cannulated forage-fed heifers supplemented or not (**CON**; *n* = 16 heifers) with a *Bacillus*-based probiotic (**BAC**; n = 16 heifers)^1^^,^^2^

Item	BAC	CON	SEM	*P*-value
**Bacterial phyla**				
** Firmicutes**	51.9	52.1	3.7	0.96
** Proteobacteria**	12.6	14.5	2.0	0.52
** Actinobacteria**	13.6	14.1	2.0	0.88
** Bacteroidetes**	10.4	11.0	2.0	0.83
** Euryarchaeota**	2.48	2.59	0.87	0.92
** Tenericutes**	4.28	1.02	2.10	0.29
** SD index**	1.50	1.48	0.06	0.84
**Bacterial genera**				
***Corynebacterium***	6.85	5.34	1.25	0.42
***Clostridium***	5.45	5.23	0.82	0.85
***Ureaplasma***	3.73	0.50	2.11	0.30
***Ruminococcus***	3.57	4.79	0.83	0.32
***Butyrivibrio***	3.09	2.93	0.70	0.87
***Bacillus***	2.90	2.28	0.63	0.50
***Blautia***	2.88	2.97	0.38	0.88
***Bacteroides***	2.85	3.15	0.58	0.72
***Caloramator***	2.62	2.44	0.59	0.83
***Methanobrevibacter***	2.56	2.83	0.97	0.85
***Turicibacter***	2.34	2.55	0.52	0.78
***Sarcina***	2.32	3.13	0.97	0.57
***Slackia***	2.08	2.44	0.55	0.65
***Mogibacterium***	2.00	1.41	0.46	0.39
***Streptococcus***	1.86	2.15	0.35	0.59
***Pedobacter***	1.44	2.11	0.52	0.39
***Prevotella***	1.40	0.894	0.49	0.48
***Oscillospira***	1.35	1.14	0.28	0.62
***Natronincola***	1.11	1.16	0.22	0.87
***Solibacillus***	0.986	1.96	1.01	0.52
** SD index**	4.36	4.48	0.10	0.43

Only bacterial phyla and genera with relative abundance above 1% are reported.

1 Heifers were assigned to receive a concentrate containing either: 1) *Bacillus licheniformis* 809 and *B. subtilis* 810 probiotic (**BAC**; 3 g/heifer daily of Bovacillus; Novonesis, Milwaukee, WI, USA) or 2) no additive (**CON**). Supplement treatments were offered from day 5 to 35 of the experiment.

2 Uterine swabs were collected on day 28, 4 h after the concentrate feeding of the day as in Moore et al. (2023).

## Discussion

This experiment evaluated BAC supplementation to growing beef cattle consuming a diet based on a C4 forage, and replicated growth rates typical of replacement heifers (Schubach et al., 2019). Supplementing BAC did not impact heifer ADG and G:F, although this experiment was not designed to assess heifer growth performance traits. Hence, heifer ADG and G:F results are presented but will not be discussed in this manuscript. Supplementing BAC also did not impact heifer feed intake, despite modulating rumen fermentation. The rumen modulatory effects of probiotics have been previously reviewed by others (McAllister et al., 2020); however, most studies have focused on high-concentrate diets and specific bacterial species, such as those producing and/or utilizing rumen lactate (Cappellozza et al., 2025). Research evaluating the effects of probiotics on nutrient fermentation and dynamics in the rumen of cattle consuming forage-based diets are limited, particularly C4 forages with elevated fiber content that impair their ruminal digestibility compared with C3 forages (Cooke et al., 2020).

Supplementing BAC reduced the proportion of acetate in the rumen fluid while increasing the proportion of propionate, butyrate, and isovalerate. Dias et al. (2022) reported that supplementing *B. licheniformis* 809 and *B. subtilis* 810 to *Bos indicus* bulls offered a byproduct-based feedlot diet reduced acetate: propionate ratio in the ruminal fluid. Molar proportions of propionate were also increased in lactating dairy cows fed *B. subtilis* in a 50% roughage diet (Sun et al., 2013). In turn, beef cows fed a forage-based diet based on C3 grass and supplemented with *B. licheniformis* 809 and *B. subtilis* 810 had similar rumen VFA concentrations and profile compared with non-supplemented cohorts (Limede et al., 2025). Supplementing BAC reduced the relative abundance of *Bacteroides*, *Pedobacter*, and *Ruminococcus* in the ruminal fluid herein, which are all bacterial genera associated with carbohydrate degradation and VFA synthesis (La Reau and Suen, 2018; Pickett et al., 2022). *Bacillus* species are capable of synthesizing fibrolytic enzymes (Schallmey et al., 2004; Luise et al., 2022) as well as expansin-like proteins that further hasten cellulose degradation in the rumen (Pech-Cervantes et al., 2019; Pan et al., 2022). Therefore, the benefits of BAC supplementation on rumen VFA profile may be related to its enzymatic activity and direct carbohydrate degradation in the rumen, resulting in less substrates for development of bacteria from the genera *Bacteroides*, *Pedobacter*, and *Ruminococcus* (Limede et al., 2025).

The effects of *Bacillus*-based probiotics on rumen liquid volume and liquid dilution rate are unknown at this point. These rumen kinetic dynamic traits are directly associated with voluntary feed intake (Allison, 1985; Allen, 2000). None of these parameters were affected by BAC supplementation in this experiment, despite treatment differences in ruminal disappearance rate and effective degradability of forage DM and NDF. More specifically, BAC supplementation improved ruminal DM and NDF disappearance rate by 26.8 and 25.8%, respectively, and improved effective degradability of forage DM and NDF by 5.2 and 3.8%, respectively. Supporting our results, others in vitro and in situ studies reported improvements in DM and NDF disappearance of forage-based substrates sampled from different regions of the globe (Pan et al., 2022; Cappellozza et al., 2023; Limede et al., 2025). Detmann et al. (2024) recently reported a positive relationship between rumen and total digestibility of NDF in C4 forages. Accordingly, BAC supplementation improved ATTD of NDF as well as DM, OM, CP, and ADF herein. Limede et al. (2025) observed greater total-tract digestibility of DM, CP, NDF, and ADF of rumen-cannulated beef cows offered diets based on C3 forages and supplemented with the *Bacillus*-based probiotic. Beef steers offered a C3 hay and ground barley supplemented with the *Bacillus*-based probiotic also had a greater total-tract DM and NDF digestibility when compared with non-supplemented cohorts (Eyre et al., 2025). These results indicate that BAC supplementation to cattle improves overall nutrient digestibility of C3 and C4 forage-based diets, which can be associated with the enzymatic activity of BAC in the rumen and throughout the gastrointestinal tract (Schallmey et al., 2004; Rhayat et al., 2019; Luise et al., 2022).

Circulating concentrations of glucose, insulin, and IGF-I are considered metabolic markers of nutrient balance in cattle (Ellenberger et al., 1989; Hess et al., 2005), suggesting improved nutritional status of BAC heifers despite similar feed intake. Blood glucose can originate from propionate produced during ruminal fermentation (Allen, 2000) and positively influences circulating concentrations of insulin (Vizcarra et al., 1998). The increase in plasma concentrations of glucose and insulin noted in BAC heifers corroborate the increased ruminal propionate proportion. Others have also reported an increase in plasma glucose concentration in forage-fed beef heifers (Izquierdo et al., 2024) and dairy cows (Arthur et al., 2025) supplemented with a *Bacillus*-based probiotic. Circulating insulin stimulates IGF-I production in the liver (Molento et al., 2002), although plasma IGF-I concentrations only differed between BAC and CON heifers on day 21 of the experimental period. Plasma urea-N is positively associated with intake of CP, rumen-degradable protein, and concentration of ruminal ammonia-N (Broderick and Clayton, 1997; Cappellozza et al., 2014a, 2014b). Heifers assigned to BAC had less plasma urea-N concentration during this experiment despite their greater ATTD of CP, and similar ammonia-N concentrations in the rumen fluid compared with CON heifers. Dias et al. (2022) reported that BAC supplementation decreased ruminal ammonia-N concentrations in feedlot bulls receiving a byproduct-based diet, but these authors did not evaluate circulating urea-N concentrations. Arthur et al. (2025) and Limede et al. (2025) also reported that BAC supplementation did not reduce ruminal ammonia-N concentrations but decreased plasma urea-N concentrations in dairy and beef cows, respectively. Perhaps BAC improved N utilization by ruminal microbes by promoting cellulolytic bacteria in the rumen such as *Bacillus* spp. (Qadis et al., 2014) as previously reported in dairy cattle (Terré et al., 2024).

To our knowledge, this is the first study evaluating the effects of BAC supplementation on the bacterial community profile of the reproductive tract of beef heifers. The main bacterial phyla and genera observed in the rumen of heifers evaluated herein agree with previous research (Khafipour et al., 2009; Ramos et al., 2021; Pickett et al., 2022), whereas the reproductive tract of healthy animals have a substantial diversity in bacterial species (Swartz et al., 2014; Clemmons et al., 2017; Ault et al., 2019a, 2019b; Quereda et al., 2020). Although these bacterial communities have many beneficial contributions to the animal, dysregulation of these communities (ie, dysbiosis) concurrent with a change in bacterial abundances may have negative consequences for fertility (Macklaim et al., 2013; Belizário and Napolitano, 2015; Green et al., 2015). *Bacillus licheniformis* and *B. subtilis* were more abundant in dairy cows without vaginal discharge associated with metritis, suggesting that these bacteria are associated with uterine health (Ballas et al., 2021). However, the authors did not report if any *Bacillus*-based probiotic was being fed, so it remains unknown if the *Bacillus* spp. are part of the normal uterine microbiota or if their presence in the uterus depends on dietary supplementation.

Treatment effects noted in vaginal microbiota profile suggest novel effects of BAC supplementation on the reproductive tract, at least in the genera levels, and support the relationship between ruminal and vaginal microbiome described by Pickett et al. (2022). The genera *Pedobacter* and *Bacteroides* were less abundant in the vagina and in the rumen of BAC heifers, although the phylum Bacteroidetes was less prevalent in the vagina of BAC heifers but not in the rumen. *Bacteroides* have been associated with endometritis in dairy cows (Bicalho et al., 2017; Cunha et al., 2018; Kudo et al., 2021) and sows (Qi et al., 2021; Zhang et al., 2021). *Corynebacterium* has been isolated from the uterus of cows without vaginal discharge (Ballas et al., 2021), and the relative abundance of this genus was also increased by 215% in BAC heifers compared with CON heifers. In turn, the uterine microbiota profile was not altered by BAC supplementation, and the relationship between ruminal and uterine microbiota was limited herein as reported by Pickett et al. (2022). The uterus is less exposed to external microbiota compared with the vagina (Sheldon et al., 2002), particularly via the cervical barrier that contributes to the limited diversity of microbes in the uterine environment (Clemmons et al., 2017). Research is warranted to further understand the benefits of feeding *Bacillus-*based and other probiotics on microbiota and overall health of the reproductive tract in cattle, which has been broadly explored in humans (Martoni et al., 2022).

In summary, feeding a *Bacillus-*based probiotic containing *B. licheniformis* 809 and *B. subtilis* 810 to beef heifers receiving a diet based on a C4 forage improved rumen fermentation traits and forage degradability, increased apparent nutrient digestibility in the whole digestive tract, and improved heifer physiological responses associated with nutritional status. Potential benefits of supplementing the *Bacillus-*based probiotic changes were observed on ruminal and vaginal microbiota profile, which provides further evidence of the link between these microbial communities in cattle. Hence, this experiment provides novel insights on how supplementing a *Bacillus*-based probiotic can enhance the utilization of C4 forage nutrients by beef heifers, with benefits to vaginal microbiota that warrant further investigation as it may lead to improved fertility.

## Abbreviations:

ADF: acid detergent fiber
ADG: average daily gain
ATTD: Apparent total-tract nutrient digestibility
BW: body weight
CP: crude protein
IGF: insulin-like growth factor
iNDF: indigestible NDF
MGA: melengestrol acetate
NDF: neutral detergent fiber
OM: organic matter
SD: Shannon diversity
VFA: volatile fatty acid
